# Application of protection motivation theory (PMT) on skin cancer preventive behaviors amongst primary school students in rural areas of Fasa city-Iran

**DOI:** 10.1186/s12885-021-09142-3

**Published:** 2022-01-03

**Authors:** Ali Khani Jeihooni, Somayeh Bashti, Bahareh Erfanian, Jeyran Ostovarfar, Pooyan Afzali Hasirini

**Affiliations:** 1 Nutrition Research Center, Department of Public Health, School of Health, Shiraz University of Medical Sciences, Shiraz, Iran; 2 Department of Anesthesiology, School of Paramedicine, Yasuj University of Medical Sciences, Yasuj, Iran; 3 Department of Public Health, School of Health, Fasa University of Medical Sciences, Fasa, Iran; 4 Department of Health Promotion, School of Health, Shiraz University of Medical Sciences, Shiraz, Iran; 5 Department of Public Health, School of Health, Kermanshah University of Medical Sciences, Kermanshah, Iran

**Keywords:** Intervention, Theory, School students, Skin cancer

## Abstract

**Background:**

Considering that exposure to sunlight in childhood and adolescence has an important role in skin cancer, so it seems that training protective behaviors in this period is more effective.

**Objectives:**

To survey the application of protection motivation theory (PMT) on skin cancer preventive behaviors among students in rural areas of Fasa city, Iran.

**Methods:**

This study was done in two stages: Phase I of this study, the descriptive-analytic and cross-sectional study was conducted in 2018 to investigate the predictive value of the protection motivation theory on skin cancer preventive behaviors. In the second stage, a quasi-experimental interventional study was conducted on 400 primary school students in 2019. The educational intervention was performed in the experimental group for 8 sessions. Data were collected using a demographic information questionnaire and protection motivation theory before and six months after the intervention.

**Results:**

The constructs of protection motivation theory predicted 58.6% of skin cancer preventive behaviors. The results indicated that there was no significant difference between the two groups in terms of knowledge, perceived sensitivity, perceived severity, reward, fear, protection motivation, response efficacy, self-efficacy, response costs, and the skin cancer preventive behaviors in before the intervention (*p* > 0.05). However, six months after the intervention, the experimental group showed a significant increase in each of the mentioned constructs and skin cancer protective behaviors (*p* < 0.05).

**Conclusion:**

This study showed the effectiveness of the intervention based on the PMT constructs in adoption of skin cancer preventive behaviors in 6 months’ post intervention in primary school students.

## Background

Cancer, particularly skin cancer, is one of the most important human health problems that annually cause many damages and imposes heavy costs on human society. Today, the role of human behaviors in the development of many health disorders including cancer, in particular skin cancer, has been proven. The National Institute of Environmental Health Sciences (NIEHS) has identified the ultraviolet light as a major contributor to skin cancer and melanoma [1, 2]. A study carried out in the United States in 2016 showed that new cases of skin cancer in women are 31,860 and deaths from this are 4320 cases per year [3]. Skin cancer is the most common cancer in the Middle East [4]. In Iran, due to the high sunlight in most seasons of the year and the lack of suitable coverings such as outdoor clothing and hats, the prevalence of skin cancer is high [5] so that skin cancer is a public health problem in the country [6]. Studies in Iran have shown that cancer is high in society [7].

Behaviors such as staying in the shade, less or no exposure to sunlight during peak hours (10 am to 3 pm), use of protective clothing such as hats with long edges and long-sleeved shirt, use of sunscreen with a SPF of 30 or higher, use of standard sunglasses, and the avoidance of artificial sources of ultraviolet light (fluorescent lamps, etc.) can be determining factors in reducing the damage caused by sunlight [8, 9].

Considering that exposure to sunlight during childhood and adolescence plays an important role in skin cancer [10], and since children and adolescents spend several hours during the week at schoolyard exposed to sunlight, schools are a good place to teach and create a pattern of health behaviors [11]. Many studies have shown that increased knowledge of the risks of skin cancer, especially in adolescents, leads to short-term improvements in skin-preventing behaviors with no long-term effects [12]. Studies have also shown that theoretical-based interventions can motivate individuals to change their attitudes and behaviors in the face of sunlight [13].

One of the most common theories of skin cancer protective behaviors is the Protection Motivation Theory (PMT), introduced by Rogers in 1975, which has since been widely accepted as a framework for predicting and intervening in hygiene behaviors [14]. PMT is organized along two cognitive mediating processes: the threat-appraisal process and the coping-appraisal process.

1. The threat appraisal assesses the maladaptive behaviors and factors affecting the possibility of engaging in potentially unhealthy behaviors; it includes internal and external rewards along with unhealthy behaviors and perception of threat (total sensitivity and perceived severity). These components increase the likelihood of an adaptive response such as reducing sunbathing or using sunscreen, while any rewards associated with continuing unprotected sun exposure (e.g. a tanned appearance) reduce this likelihood [15, 16].
2. Coping appraisal assesses the ability to cope with and respond to a threatened risk. It includes the perceived response efficacy, perceived self-efficacy and response costs. The response efficacy is the expectation of the person that the adaptive response (self-protection) can eliminate the threat, and the effect the proposed preventive behavior is expected to increase the response [16]. The self-efficacy is a person’s belief in his/her ability to successfully perform the adaptive and prescribed behaviors. The perception of high self-efficacy is expected to lead to more positive responses in the individual [16, 17]. Response costs are any costs (e.g., monetary, personal, time, effort) associated with taking the adaptive coping response. Increasing the cost of using prescribed health behaviors reduces the motivation to behave [15]. Response efficacy and self-efficacy will increase the probability of selecting the adaptive response, whereas response costs will decrease the probability of selecting the adaptive response [15]. The effectiveness of the two cognitive mediational processes leads to the formation of protection motivation and behavior [16]. In order that the protection motivation to be recalled, perceived severity and perceived sensitivity must overcome the maladaptive response rewards (no self-protection); and perceived self-efficacy and perceived response efficacy must overcome adaptive response costs (self-protection). Protection motivation is an intermediate variable between the stages of threat appraisal, coping appraisal and preventive behavior (protective behavior) [17].

Despite the increasing knowledge of the general population, the level of knowledge of individuals about the effects of choosing and the need to carry out preventive behaviors of skin cancer, especially in rural communities of Iran, is not acceptable and, compared with other health problems, it didn’t attract much attention. Due to the vulnerability of students, especially rural students, and since there has not been a study on primary school students in rural areas of Fasa, the aim of this study was to investigate the effect of educational intervention using the theory of protective motivation on preventive behaviors of skin cancer among students in rural areas. Fasa city, Fars province, Iran.

## Methods

### Research design

The present study was conducted in two stages. First, a cross-sectional descriptive-analytic study was carried out in 2018 to investigate the predictive value of the protection motivation theory on skin cancer preventive behaviors on randomly selected 400 students from the fifth-grade primary school in Fasa, Iran.

### Participants

According to the results of similar studies [18–20], and considering 95% confidence level, d = 0.05, and *p* = 0.65, the sample size was estimated to be 350. To ensure accuracy, the data were gathered from more than 400 students. Sampling method was random cluster sampling. Thus, one boy’s primary school, one girl’s school from district 1 and one boy’s primary school, one girl’s primary schools from district 2 were randomly selected (the students of the selected schools had almost similar demographic, economic, and social characteristics).

On the basis of the review of similar texts [21], with a 95% confidence coefficient and 80% power of the test, and taking into account the loss of samples, the sample size was determined 400 and divided into two equal groups of experimental and control (200 in each of the experimental and control groups). In order to select the samples, out of 32 girls ‘and boys’ primary schools in Fasa, eight schools were randomly selected (four primary schools as the experimental group and four primary schools as the control group), 50 students (fifth grade) were selected from each elementary school. Samples should enroll in one of the state-run primary schools and voluntarily entered the study.

Inclusion criteria included; Fifth grade students from elementary schools and regular attendance at school, and exclusion criteria included; parents and students’ unwillingness to participate further and nonattendance in at least two educational sessions) Because of the researcher’s relationship with the students, all students completed the questionnaires (.

To comply with ethical considerations, while obtaining permission from Fasa University of Medical Sciences Ethics Committee and Fasa Department of Education, briefing the parents and students, and gaining their acceptance (Due to the fact that the study was conducted in a school environment and the researchers had obtained the necessary permits from the responsible authorities, the parents of the students agreed to conduct the study with the participation of their children); goals, importance, and necessity of conducting the research project were repeated to them, and the samples were assured that the information would be treated strictly confidential.

### Instruments

PMT variables were measured trough a designed and validated questionnaire by Dehbari et al. [18], Khani Jeihooni and Moradi [21] and Khorsandi et al. [22] for assessing the PMT variables in terms of the sun protective behaviors for prevention of skin cancer among primary school students. The validity and reliability of the instrument has been confirmed in the mentioned studies so that in order to determine the face validity of the instrument, a list of compiled items has been completed by 50 primary school students with similar demographic, economic and social characteristics. In order to determine the validity of the content, the opinions of 12 experts and experts (outside the research team) in the field of health education and health promotion (10 people) and dermatologist (1 person) and occupational health (1 person) have been used. The overall reliability of the research tool by calculating Cronbach’s alpha was 0.88. reliability of the knowledge questionnaire 0.89, perceived sensitivity 0.86, perceived intensity 0.88, reward 0.87, fear 0.78, protection motivation 0.84, response efficiency 0.77, self-efficacy 0.89, response costs 0.80 and behavior 0.86 has been confirmed.

This tool included demographic information (age, gender, household size, parents’ education, parents’ occupation) and a questionnaire based on the protection motivation theory.

The questions of the constructs of the protection motivation theory included perceived sensitivity (5 questions, score range of 5–25) for example, “skin cancer may be observed only in people who have white skin”, perceived severity (5 questions, score range of 5–25), for example “skin cancer can be deadly”, reward (5 questions, score range of 5–25), for example “I feel good when I am in the sun” fear (5 questions, score range of 5–25), for example “I’m afraid of thinking about skin cancer” protection motivation (6 questions, score range of 6–30), for example “I motivate to spend less time outdoors” response efficacy (5 questions, score range of 5–25), for example “using sunglasses will help in preventing skin cancer” self-efficacy (6 questions, score range of 6–30), for example “I can use sunscreen consistently before I go outside” response costs (5 questions, score range of 5–25), for example “sunscreen is expensive” knowledge questions (20 questions, score range of 0–20),” Risk factor; Having dark colored skin?” and skin cancer preventive behaviors (10 questions - scores range of 10–50) for example “do you wear hat with a brim when you are in the sun?”. The constructs of the protection motivation theory, based on the Likert 5-point scale, included ‘agree’ or ‘disagree’ choices with the score of 1 to 5. Knowledge questions were in the form of multiple-choice, the correct choice scored 1 and the wrong choice scored zero. To measure skin cancer preventive behaviors, the Likert 5-point scale (from never to always) was used.

### Procedure and program

After the selection of the experimental and control groups, the purpose of the research and how to do the work was explained to every student and their parents, as well as the authorities of schools and teachers. The pre-test questionnaire was completed by the experimental and control groups. According to the pre-test results and the importance of predictors, the educational content was based on the theory of protection motivation. Educational intervention for the experimental group consisted of 8 sessions of 50–55 min in lecture, question and answer, group discussion, using posters and educational pamphlets, cartoon films, and PowerPoint presentations. The sessions were held weekly in one of the classes. Also, intervention sessions were held at school time instead of health class sessions, so all students were present in the class and there was no falling in sample size in the study. At the end of the educational sessions, participants were given a complete educational package, plus a gift, and a notebook. The details of the training sessions are presented in Table 1.

**Table 1:** The details of the training sessions

Sessions	Details	Learning goals	Presentation	Presenter
*First Session*	Skin cancer, symptoms, complications, and diagnosis were introduced	Increase awareness and knowledge	lecture, group discussion, cartoon films	Dermatologist
*Second Session*	The prevalence of skin cancer and risk factors and being in exposure of sunlight was explained	Increase knowledge, create sensitivity and severity in students about skin cancer	video clips and PowerPoint presentations	Dermatologist and PhD in health education and promotion
*Third Session*	changes in everyday behaviors such as the avoidance of intense sunburn (10 am to 4 pm), wearing thick clothing when exposed to sunlight, using sunscreen with a SPF of 30 or higher	Motivate students to protect their skin	question and answer	PhD in health education and promotion
Forth session	the avoidance of artificial sources of ultraviolet light, using ultraviolet protected glasses. use of skin cancer preventive tools, and personal protective equipment.	students learn ways to protect their skin and motivate them to apply strategies	group discussion, cartoon films	PhD in health education and promotion
Fifth session	The benefits of prevention behaviors from skin cancer were emphasized and demanded information and facilities were provided for subjects. This session was held with the presence of teachers, school officials, a family member, and staff of health care canters and their role in prevention of skin cancer.	The rewards of protective behaviors were expressed to students to increase their motivation to engage in protective behaviors	group discussion, video clips and PowerPoint presentations	Dermatologist, health center officials and a family member
Sixth session	Skin cancer and its risk factors, symptoms, and complications. Barriers and costs can reduce the choice of skin preventive behaviors	Assess students’ perceived sensitivity and severity to skin cancer in group discussions and find solutions to barriers and costs of choosing protective behaviors	group discussion, using posters and educational pamphlets	A person suffering from skin cancer
Seventh session	Ways of increasing self-efficacy in subjects for protection of sun, using sunscreen with a SPF of 30 or higher, the avoidance of artificial sources of ultraviolet light, using ultraviolet protected glasses and personal protective equipment	Increasing students ‘self-efficacy in performing skin protection behaviors, increasing students’ awareness of the effectiveness of protective behaviors	group discussion, video clips and PowerPoint presentations	PhD in health education and promotion
Eighth sessions	Previous sessions were reviewed and educational booklets were given to subjects	Ensure the impact of educational intervention using group discussion and question and answer from research participants	group discussion, question and answer	PhD in health education and promotion

At the end of the sessions, they were given a booklet. The students were divided into groups of 10, and groups of friends and co-workers were formed. For controlling activities, an educational session was monthly held for students and a WhatsApp Group was formed for the parents of students to exchange information. Within two months and four months after the intervention, two follow-up sessions were held for students. Six months after the intervention, both groups (experimental and control) completed the questionnaire. For the control group, an animation about skin-preventing behaviors was also presented at the end of the study.

### Statistical analysis

The data were analyzed using spss22 software and logistic regression statistical test, Pearson correlation coefficient, paired t-test, independent t-test, and chi-square test. The significance level was considered 0.05.

## Results

Four hundred students from the fifth-grade primary school ​that 135(33.75%) were male and 265(66.25%) were female, with a mean age of 11.11 ± 0.79 participated in the cross-sectional study. In terms of students’ parental education status, 17.75% of fathers had academic education and 84.75% of mothers had undergraduate education. Most fathers (72.75%) were self-employed and the majority of students stated that their mothers (80.6%) were housewives. Also, the mean of household size was 4.82 + 1.16.

The results showed that there was a significant positive relationship between the skin cancer preventive behaviors and perceived sensitivity (r = 0.178, *p* = 0.007), perceived severity (r = 0.124, *p* = 0.016), protection motivation (r = 0.184, *p* = 0.004), response efficacy (r = 1.20, *p* = 0.022), self-efficacy (r = 0.180, *p* = 0.018), fear (r = 0.144, *p* = 0.009), and knowledge (r = 0.102, r = 0.037); also, there was a significant negative relationship between the skin cancer preventive behaviors and rewards (r = −0.126, *p* = 0.025),, and response costs (r = −0.115, and *p* = 0.016).

Logistic regression analysis results are presented in Table 2 to predict skin cancer preventive behaviors based on the protection motivation theory. According to the findings, protective motivation, self-efficacy, and perceived sensitivity were the strongest predictors of skin cancer preventive behaviors. In general, the studied variables predicted 58.6% of behavior.

**Table 2:** Regression analysis of factors related to skin cancer protective behaviors among students

Variables	Beta	B	P	Dependent variable
Perceived sensitivity	0.206	0.142	0.036	
Perceived severity	0.187	0.135	0.039	
Reward	−0.118	−0.123	0.047	
Fear	0.126	0.128	0.044	
Protection motivation	0.228	0.165	0.014	Skin cancer protective behaviors
Self-efficacy	0.219	0.156	0.025	R^2^ = 0.586
Response efficacy	0.089	0.068	0.033	R^2^Adjusted = 0.038
Response costs	−0.078	−0.065	0.037	
Knowledge	0.125	0.094	0.048	

In the semi-experimental study, the mean age of the students in the experimental and control groups was 11.26 ± 0.72 and 11.18 ± 0.80 years, respectively (*p* = 0.286); the mean of household size in the experimental group was 4.9 ± 1.0 and in the control group 4.24 ± 1.20 (*p* = 0.222), which, based on independent t-test, did not show a significant difference in the age of the two groups. The results of this study showed that the Chi-square test showed no significant difference between the two groups in terms of gender and parents’ occupation and education (Table 3).

**Table 3:** Comparison of demographic variables of students in the experimental and the control groups

Variable		Experimental group N = 200		Control group N = 200		Significance level
		Number	Percent	Number	Percent	
Gender	Female	108	54	112	56	0.197
	Male	92	46	88	44	
Father’s occupation	Employed	78	39	82	41	0.262
	Self-employed	122	61	118	59	
Mother’s occupation	Employed	42	21	38	19	0.257
	Housewife	158	79	162	81	
Father’s educational level	Illiterate	2	1	4	2	0.163
	primary school	28	14	32	16	
	middle school	50	25	44	22	
	High school	84	42	76	38	
	Academic	36	18	44	22	
Mother’s educational level	Illiterate	4	2	6	3	0.178
	primary school	30	15	36	18	
	middle school	60	30	56	28	
	High school	86	43	78	39	
	Academic	20	10	24	12	

The results also showed that there was no significant difference between the two groups in terms of knowledge, perceived sensitivity, perceived severity, reward, fear, protection motivation, response efficacy, self-efficacy, response costs, and the skin cancer preventive behaviors before the intervention; but, six months after the educational intervention, there was a significant difference and the paired t-test showed that the mean score of knowledge, perceived sensitivity, perceived severity, fear, protection motivation, response efficacy, self-efficacy, and the skin cancer preventive behaviors significantly increased in the experimental group. Furthermore, the mean score of rewards and response costs decreased. However, no significant change was observed in the control group. (Table 4).

**Table 4:** Comparison of the mean score of PMT variables and Skin cancer protective behaviors in the experimental and the control groups before the intervention and six months after the educational intervention

variable	Group	Before intervention M ± SD	Six months after intervention MM ± SD	Paired t-test
knowledge	experimental	6.13 ± 2.26	> 0.001p	*p* > 0.001
	control	6.45 ± 2.03	44.12 ± 3.25	*p* = 0.172
	Independent t-test	0.184	19.01 ± 3.64	
perceived sensitivity	experimental	7.20 ± 2.32	*p* > 0.001	*p* > 0.001
	control	7.21 ± 2.24	8.07 ± 2.16	*p* = 0.185
	Independent t-test	0.166	*p* > 0.001	
perceived severity	experimental	6.75 ± 2.39	22.07 ± 2.14	*p* > 0.001
	control	6.46 ± 2.54	7.18 ± 2.40	*p* = 0.192
	Independent t-test	0.189	*p* > 0.001	
reward	experimental	21.15 ± 2.46	12.04 ± 2.25	*p* > 0.001
	control	20.37 ± 2.75	19.89 ± 2.39	*p* = 0.181
	Independent t-test	0.156	*p* > 0.001	
fear	experimental	5.87 ± 2.97	18.28 ± 2.18	*p* > 0.001
	control	6.04 ± 2.72	6.21 ± 2.44	*p* = 0.178
	Independent t-test	0.201	*p* > 0.001	
protection motivation	experimental	13.43 ± 2.68	25.77 ± 2.68	*p* > 0.001
	control	14.02 ± 2.49	15.12 ± 2.44	*p* = 0.172
	Independent t-test	0.196	*p* > 0.001	
response efficacy	experimental	7.25 ± 2.18	20.32 ± 2.19	*p* > 0.001
	control	7.80 ± 2.03	8.41 ± 2.10	*p* = 0.155
	Independent t-test	0.164	*p* > 0.001	
self-efficacy	experimental	10.21 ± 2.08	26.10 ± 2.12	*p* > 0.001
	control	10.83 ± 2.06	11.09 ± 2.19	*p* = 0.190
	Independent t-test	0.191	*p* > 0.001	
response costs	experimental	19.76 ± 2.57	7.15 ± 2.06	> 0.001p
	control	18.89 ± 2.84	18.12 ± 2.35	= 0.174p
	Independent t-test	0.124	> 0.001p	
Skin cancer protective behaviors	experimental	18.44 ± 3.58	44.12 ± 3.25	> 0.001p
	control	17.92 ± 3.82	19.01 ± 3.64	= 0.168p
	Independent t-test	0.186	> 0.001p	

## Discussion

In this research, a cross-sectional study was conducted to identify the predictors of the protection motivation theory on 400 primary school students. The results showed that there was a significant positive relationship between skin cancer preventive behaviors and perceived sensitivity, perceived self-efficacy, perceived severity, protection motivation, response efficacy, and knowledge. However, there was a significant negative relationship between skin cancer preventive behaviors and the rewards and response costs. This finding is quite similar with those found by Zare Sakhvidi et al. [23].

Overall, the studied variables predicted 58.6% of the variance of the skin cancer preventive behaviors; protection motivation, self-efficacy, and perceived sensitivity were the strongest predictors. In a study by Babazadeh et al. and Zare Sakhvidi et al. perceived sensitivity, reward, self-efficacy and response costs were the predictors of skin cancer preventive behaviors [20, 23]. In the study of Werk et al., knowledge and self-efficacy were predictors of the skin cancer preventive behaviors [24]. In the study of Hosseini et al. the constructs of the protection motivation theory could predict 32.6% of the variance of protective behaviors, that perceived sensitivity, perceived reward and fear constructs were the strongest predictors, in which the perceived sensitivity was the strongest [25]. These results can indicate that the more vulnerable a person is to health risks (skin cancer and sun damage), the more likely he or she is to engage in skin-protecting behaviors, Therefore, he is more likely to engage in protective behaviors. Also, the more a student believes that he/she can perform sunscreen behaviors, the more he/she intends to do those behaviors.

The results of the quasi-experimental study, showed that before the intervention, the mean score of knowledge in both the experimental and control groups was low, but in the six months after the educational intervention, the knowledge of the subjects in the experimental group significantly increased, while in the control group did not change significantly. The results of Loescher et al. study showed that high school students significantly improved skin cancer prevention knowledge scores and self-reported skin cancer prevention behavior over 3 to 4 months post training and intervention implementation [26]. The reason for increasing knowledge can be the intervention group’s access to new information and participation in training classes held by researchers.

The findings of this study showed that there was no significant difference between the two groups of intervention and control regarding the mean score of perceived sensitivity construct before the intervention, while the mean score of perceived sensitivity after 6 months of the intervention was significantly higher in the experimental group compared to the control group. This means that after the intervention, most of the students in the intervention group believed that they were at risk for skin cancer. The results of Babazadeh et al. [27], Sadeghi et al. [28], and Malmir et al. [29] were consistent with the findings of this study. According to the results of the study which show that the intervention increased students’ awareness of skin cancer, increasing the mean score of sensitivity perceived in students seems reasonable.

The mean score of perceived severity in the experimental group was significantly increased in the six months after the intervention, while there was no significant change in the control group. The findings of this study were consistent with the results of other studies [27, 28, 30–33]. These results indicate that when people are aware of the side effects of sunlight, they are more likely to do protective behaviors. Therefore, in educational interventions, one can benefit from the experiences of those who have been affected by sunlight to increase their perceived severity.

The findings of this study showed that the internal and external rewards decreased significantly in the experimental group in the 6 months after intervention in comparison with the control group. The results of Baghiani moghadam et al. [31], Babazadeh et al. [27], Taheri et al. [34] and Afshari et al. [4] were consistent with the results of this study. A significant negative relationship between the rewards and the skin cancer preventive behaviors in the cross-sectional study and the reduction of the reward score after the intervention means that the more the internal and external rewards of the maladaptive behaviors, the less probable doing preventive behaviors. Therefore, in educational interventions, the disadvantages of maladaptive behaviors and the benefits of appropriate skin cancer preventive behaviors should be explained to the subjects.

The findings of this study showed a significant increase in the mean score of the fear construct in the 6 months after intervention in the intervention group, while there was no significant change in the control group. The results of studies by Taheri et al. [34], Afshari et al. [4], Babazadeh et al. [27], and Khosravi et al. [35] were consistent with the present study. It seems in this educational program, fear of illness and other related problems, including fear of loss of appearances, rejection by the community, anxiety, depression, and the fear of the destructive effect of cancer, affected student’s motivation for practicing preventive behaviors against sunlight.

In this research, the mean score of response costs for skin cancer preventive behaviors among students in the experimental group significantly decreased after the intervention. The results of Sadeghi et al. showed that by removing barriers, the health behaviors increase [28]. The results of other studies were consistent with the findings of this study [27, 34, 36], However, in Maseudi et al., The mean score of response costs did not show a significant increase after the intervention [37]. Response costs could be a barrier to the skin cancer preventing behaviors, so educational programs and guiding people would remove barriers and increase preventive behaviors.

The results of this study showed that the mean score of perceived response increased significantly in the experimental group after the intervention, while no significant changes were observed in the control group. The findings of this study were consistent with other studies [27, 34, 38, 39]. So, the intervention can enable students to respond appropriately and practice protective behaviors based on recommendations to eliminate skin cancer threats.

The mean score of perceived self-efficacy in both experimental and control groups was low at the pre-intervention stage but in six months after the intervention, there was a significant increase in the experimental group, while there was no significant change in the control group. The findings of this study were consistent with the studies by Taheri et al. [34], Zare Sakhvidi et al. [23], Werk et al. [24], and Craciun et al. [40]. Self-efficacy is the ability of students to do sunlight prevention behaviors; thus, an educational program can help them to identify and apply their ability to use sunlight protection products.

The present study showed that there is a significant difference in the score of the protection motivation construct between the experimental and control groups in the 6 months after the intervention. The results of Prentice-Dunn et al.’s study showed that intervention increased the intention to protect against skin cancer in the intervention group, as compared with the control group (5). Also, the results of other studies were consistent with the findings of this study [22, 31, 35–37, 41, 42]. Protection motivation was influenced by other constructs of the motivation protection theory so that with the increase in the mean score of the constructs of this theory, the protection motivation also increases.

In the six months after the intervention, the experimental group showed a significant increase compared to the control group. Compared to the control group, using sun protection gears (sunscreen, gloves, caps, etc.) in the experimental group after the intervention was significantly higher than before. The result of Çelik et al. study, among nursing students, the percentage of “negative behaviors” in response to skin cancer protective behaviors was higher than for “positive behaviors” [43]. In studies of Hernandez et al. [44], Nadrian et al. [45], Loescher et al. [26], Sumen et al. [9] Khani Jeihooni and Moradi [21], consistent with the results of this study, educational intervention contributed to the promotion of the skin cancer preventive behaviors. This increase indicates the effectiveness of the educational intervention. This means that when people feel susceptible to a health risk, fear raises in them, and they see themselves as fit for the proposed behavior, following a reward of adaptive behavior (protective measures). As a result, their motivation or intention to behavior increases.

One of the strengths of this study is the cross-sectional study, followed by an educational intervention based on the results of a cross-sectional study (prerequisites). A study on a susceptible group such as rural students, regarding the tropical region of Fasa and the agricultural profession in this area, is another strength of the present study. The limitations of this study include the collection of information through the self-reporting method.

## Conclusion

The results of this study showed that as the level of knowledge, perceived threats, protection motivation, fear, response efficacy, and self-efficacy increase; and perceived rewards and response costs decrease, the skin cancer preventive behaviors enhance. Therefore, the protection motivation theory is recommended as a suitable model for promoting skin cancer preventive behaviors. Given the susceptibility of children and adolescents to skin cancer, the need to provide educational programs in this area is quite tangible. Since schools are a good place to educate and create patterns of health behaviors, the Department of Education should have a special focus on informing students about the prevention of skin cancer. Supporters such as the family, school officials and teachers have a role to play in promoting these behaviors that can be achieved with appropriate strategies.

## Data Availability

The datasets used and/or analyzed during the current study are available from the corresponding author on reasonable request.

## Abbreviation

PMT: Protection Motivation Theory
